# High Occurrence of Emerged *Lophomonas* Infection among Patients Suspected of Having Pulmonary Tuberculosis: In-House PCR-Based Evidence

**DOI:** 10.1155/2022/2742164

**Published:** 2022-12-01

**Authors:** Hamed Kalani, Ayeneh Pangh, Maryam Nakhaei, Hajar Ziaei Hezarjaribi, Mahdi Fakhar, Ali Sharifpour, Elham Sadat Banimostafavi, Rabeeh Tabaripour

**Affiliations:** ^1^Infectious Diseases Research Center, Golestan University of Medical Sciences, Gorgan, Iran; ^2^Iranian National Registry Center for Lophomoniasis (INRCL), Imam Khomeini Hospital, Mazandaran University of Medical Sciences, Sari, Iran; ^3^Pulmonary and Critical Care Division, Imam Khomeini Hospital, Iranian National Registry Center for Lophomoniasis (INRCL), Mazandaran University of Medical Sciences, Sari, Iran; ^4^Department of Radiology, Imam Khomeini Hospital, Mazandaran University of Medical Sciences, Sari, Iran

## Abstract

**Objectives:**

*Lophomonas* infection is a respiratory disease in humans that is associated with symptoms of cough, sputum, dyspnea, and sometimes hemoptysis, which shows the importance of differentiating this disease from tuberculosis and asthma.

**Methods:**

This study was performed on 216 participants suspected of having tuberculosis who had symptoms of fever, chronic cough, or sputum and were referred to tuberculosis laboratories in three cities in Golestan Province, northeastern Iran, during 2019-2020. A sputum sample was taken from the suspected patients. DNA was extracted from the frozen samples, and an in-house polymerase chain reaction was performed to detect the *Lophomonas* DNA.

**Results:**

Out of 216 subjects, 47 (21.75%) were infected with *Lophomonas* spp. Moreover, 9 patients (4.2%) were infected with tuberculosis. Also, 2 patients had a comorbidity of tuberculosis and *Lophomonas* infection (*P*=0.63). There was no significant difference in the comparison of symptoms and the rate of *Lophomonas* infection (*P*=0.84), but in the comparison of the set of symptoms of cough, sputum, and fever with those of cough and sputum, cough with fever, sputum with fever, and the rate of *Lophomonas* infection, there was a significant difference (*P*=0.012).

**Conclusions:**

*Lophomonas* infection was relatively high in patients suspected of having tuberculosis and due to the similar clinical symptoms of *Lophomonas* infection and tuberculosis; it is recommended that the sputum samples of subjects suspected of having tuberculosis be examined for this parasite in order to make a correct diagnosis and the patients receive timely treatment and the appropriate medication.

## 1. Introduction

Tuberculosis (TB) is an important infectious disease and one of the top 10 causes of death in the world. In 2019, TB killed 1.4 million people, 208,000 of whom were HIV-positive. TB has been reported in all countries and age groups [1]. According to the World Health Organization (WHO) report in 2019, the incidence rate of TB was <10 cases per 100,000 subjects in Iran [1, 2]. Golestan Province, in northeastern Iran, is the second most infected province in terms of cases of TB in Iran [3, 4].

The emergence of lophomoniasis, caused by the protozoan *Lophomonas* spp., mainly as a commensal inhabitant of cockroaches, is an unusual cause of bronchopulmonary infections [5]. The flagellated parasite found in airway samples aids in diagnosis, though their morphology is identical to ciliated bronchial epithelium, which makes interpretation difficult. The dust contaminated with cysts may be inhaled, initiating infection. Infections are reported mostly in adults; very few have been described in children [6].

*Lophomonas* infection is a respiratory disease in humans that is associated with symptoms of cough, sputum, dyspnea, and sometimes hemoptysis, which shows the importance of differentiating this disease from TB and asthma [7, 8]. So far, human *Lophomonas* infection has been reported from two Iranian provinces in northern (Mazandaran, Sari) and eastern (Khorasan Razavi, Mashhad) Iran [9–11]. According to the information published by the Iranian National Registry Center for Lophomoniasis (INRCL), *Lophomonas blattarum* (L. *blattarum*) has been confirmed by molecular and sequencing techniques in Iran [1]. Also, the parasite was isolated recently from German cockroaches (*Blattella germanica*) in Mazandaran, northern of Iran [6].

Till date, no study has been performed on patients suspected of having TB, and only case reports of *Lophomonas* and TB coinfection have been published around the world. Therefore, considering the similarity of tuberculosis with lophomoniasis in terms of clinical patterns and the lack of data about the status of this infection in patients suspected of pulmonary tuberculosis, the present study attempts to investigate the status of *Lophomonas* infection in these individuals using an in-house PCR test.

## 2. Methods

### 2.1. Study Area

**Figure 1:**
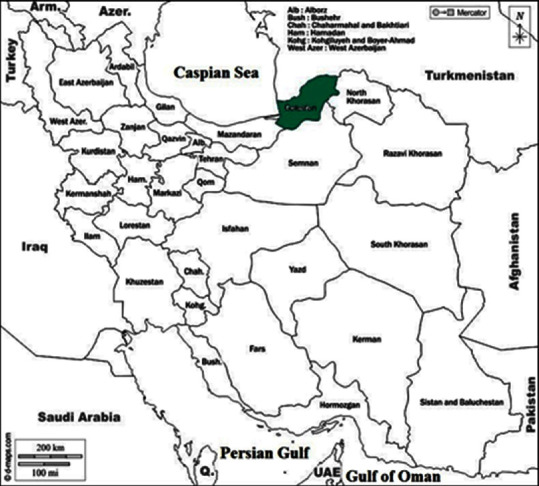
The map of Golestan Province, northeastern Iran.

Golestan Province is one of the northern provinces of Iran. Its area is 20,367 km^3^. The province has seven counties. The population of the province in the general census of population in 2016 was 1,868,819 subjects. This province has a diverse climate due to its special geographical location. Golestan Province is divided into the following three areas in terms of unevenness: mountainous area, foothill area, and plain area.  Figure 1 shows a map of Golestan Province.

### 2.2. Participants and Sampling

216 participants were randomly selected from patients with suspected tuberculosis who had symptoms of fever, chronic cough, or sputum and were referred to tuberculosis laboratories in three cities of Golestan Province, including Aq-Qala, Gonbad-e Kavus, and Gorgan in the northeastern of Iran, during November 2019–September 2020. All subjects entered the study with informed consent, and for each of them, a questionnaire containing the studied variables was considered. The study was confirmed by the Ethical Committee of Mazandaran University of Medical Sciences (ethical code: IR.MAZUMS.REC.1399.6901).

At the tuberculosis diagnostic centers, a sputum sample was taken from the patients for testing, and we transferred a part of this sample to the laboratory for testing of the *Lophomonas* parasite. Since the samples were suspected of having tuberculosis, the study was conducted on them in compliance with all hygienic points. The samples were stored at −20°C until use.

### 2.3. DNA Extraction

To genomic DNA extraction, frozen sputum specimens (about 1 ml) were thawed at room temperature and then spin at 500 g for 5 min [11]. Next, the sediment was used for DNA extraction. Lysis buffer (0.93 g of EDTA, 0.5 g of SDS, 1.43 g of NaCL, and 3.15 g of Tris-HCL in 100 ml of distilled water) was added and mixed with each of the sputum sediments at an equal volume, and the microtubes were vortexed twice for 10 seconds each time, then incubated at 60°C for 30 min, centrifuged at 4,000 × g, 4°C, for 10 min, and the supernatants were transferred to the new microtubes. 15 *μ*L of proteinase *K* (20 mg/ml) was added to each of the supernatants, which were incubated at 60°C for 45 min, and then 200 *μ*L of phenol: chloroform solution (1 : 1; w/v) was added to each of the microtubes, and centrifuged at 14,000 × g, 4°C, for 15 min. 400 *μ*L of absolute ethanol was added to the separated supernatants, vortexed once, and put into the microtubes at −20°C for 2 hours. The samples were centrifuged at 14,000, 4°C, for 15 min and the pellets containing DNA were harvested and kept at −70 until use.

### 2.4. Conventional Genus-Specific PCR (In-House PCR)

Genus-specific PCR was performed to detect the DNA of the *Lophomonas* parasite in the extracted sputum samples as described by Fakhar et al. [12]. The used primers were as follows: forward 5′-GAGAAGGCGCCTGAGAGAT-3′ and reverse 5′-ATGGGAGCAAACTCGCAGA-3′. 1 *μ*L of each primer and 12.5 *μ*L of mastermix were added to a microtube, and the volume was adjusted to 25 *μ*L with ddH_2_O. The time and temperature of the reaction were: initial denaturation at 94°C for 4 min followed by 35 cycles of denaturation at 94°C for 30 s, annealing at 55°C for 45 s, extension at 70°C for 30 s, and final extension of DNA for 5 min. The samples after PCR were run on a 2% agarose gel in a Tris-boric acid-EDTA solution (w/v). The gel was assessed under a UV transilluminator after staining with a SafeView^TM^ DNA Stain. A 214 bp band was related to *Lophomonas* spp. In the PCR processing, L. *blattarum* (Accession Numbers: MZ093070) was used as positive and distilled H_2_O and DNA of *Trichomonas vaginalis* as a negative control.

### 2.5. Data Analysis

For qualitative variables, we used percentage and frequency. A *P* value of less than 0.05 was regarded as statistically significant. Demographic data were analyzed by IBM SPSS version 26.

## 3. Results

### 3.1. Demographic and Clinical Characteristics of Participants

216 subjects participated in the study, of whom 85 (39.3%) were from Aq Qala, 103 (47.7%) from Gorgan, and 28 (13%) from Gonbad-e Kavus districts. The lowest age of the participants in this study was 8 years, and the highest was 83 years. 99 (45.8%) of the participants were female and 117 (54.2%) were male. In terms of clinical symptoms, 5 subjects (2.3%) had no symptoms. 43 patients (19.9%) had fever, cough, or sputum. 111 subjects (51.4%) had two symptoms, and 57 patients (26.4%) had all three symptoms (Table 1).

### 3.2. Positive Cases of *Lophomonas* Infection and Tuberculosis

**Figure 2:**
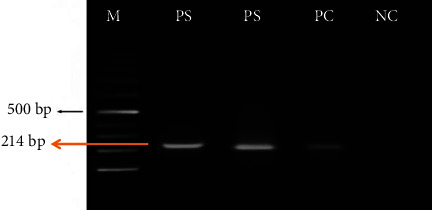
A 214 bp band from the PCR products of positive sputum samples infected with *Lophomonas* spp., in 2% agarose gel electrophoresis. *M* = marker (100 bp). PS = positive samples. PC = positive control (L. *blattarum*; accession numbers: MZ093070). NC = negative control (distilled H_2_O).

Out of 216 subjects, a total of 47 (21.75%) were infected with *Lophomonas* spp., (Table 1). Nine subjects (4.2%) were infected with tuberculosis. Of them, 3 (3.53%) were from Aq Qala, and 6 (5.82%) were from Gorgan districts. Also, 2 patients had a comorbidity of tuberculosis and *Lophomonas* infection (*P*=0.63). One of them was from Aq Qala, and the other was from Gorgan City. The most clinical symptom of the patients was fever, following sputum and cough, respectively. There was no significant difference in the comparison of clinical symptoms separately and the rate of *Lophomonas* infection (*P*=0.84; Table 1), but in the comparison of the set of symptoms of cough, sputum, with fever, cough with sputum, cough with fever, sputum with fever, and the rate of *Lophomonas* infection, there was a significant difference (*P*=0.012). The result of PCR is shown in Figure 2.

## 4. Discussion

So far, *Lophomonas* spp., has been mostly isolated from sputum and BAL fluid clinical samples [5]. For this reason, in the present study, we used sputum samples to examine *Lophomonas* infection.

Currently, the Giemsa, Trichrome, and Papanicolaou staining methods are used to identify *Lophomonas* parasites [13], but the identification of this parasite is difficult to detect based on morphology due to its high similarity to normal and/or atypical lung epithelial cells [14]. Therefore, the PCR method increases sensitivity and specificity in the diagnosis of this parasite [12]. The results of the present study showed that 47 out of 216 cases (21.76%) were infected with the parasite by PCR. Considering the emerging nature of this infection and the lack of evidence of the prevalence of *Lophomonas* infection in people suspected of tuberculosis, the results of the study cannot be compared with other studies, and the results of our study are the first scientific evidence in this regard.

To date, accurate data on the prevalence of this parasite in different countries are not available, and most studies have been performed in the form of case reports or case series of infected subjects, and most of them are from Asia [5, 14, 15]. However, the real global burden of *Lophomonas* infection is unknown; the most cases have been recorded in Iran and China [13]. Based on a systematic review regarding global *Lophomonas* infection the coexistence of tuberculosis with lophomoniasis was relative high (11.4%; 17/149) [14].

A study showed that the coexistence of tuberculosis and lophomoniasis was relatively common (11.4%; 17/149) [14]. According to the high prevalence (21.75%) of *Lophomonas* infection among the participants suspected of having tuberculosis in the present study, Golestan Province could be considered as one of the endemic areas for the emerged protozoan parasite.

Consistent with some evidence, *Lophomonas* infection is not affected by age or gender [5, 11, 16], and the findings of our study support this (Table 1). However, the majority of cases of infection were observed in subjects over 50 years old, which could be attributed to the large sample size in this age group, the possibility of more parasite exposure for these subjects, the occurrence of clinical symptoms in old age, and more doctor visits.

Few studies have examined the association of *Lophomonas* infection with other respiratory diseases, including pneumonia, chronic obstructive pulmonary disease, lung cancer, allergies, interstitial lung disease, pulmonary abscess, tuberculosis, chronic cough, respiratory cysts, asthma, and bronchitis [5, 11, 15, 16]. Of these, 21 cases of coinfection with lophomoniasis and tuberculosis have been reported so far [5, 8, 11, 17]. However, in the present study, we showed that there is no significant relationship between lophomoniasis and tuberculosis (*P*=0.63) and only in two cases with lophomoniasis and tuberculosis coinfection were observed. Interestingly, 47 cases of *Lophomonas* infection were observed in 216 patients suspected of having tuberculosis, indicating the importance of examining this parasite in subjects who exhibit the set of symptoms of cough with sputum and with or without fever.

The most important clinical signs that suggest the possibility of *Lophomonas* infection include cough, sputum, and fever, which are observed in patients with asthma or tuberculosis, so researchers have shown that there is a significant relationship between the symptoms of asthma and *Lophomonas* infection [11, 18–20]. Recent studies have noted that asthma-like symptoms in patients with *Lophomonas* infection are milder than those in subjects with asthma.

According to the results of a study, the most important symptoms in children and adults with *Lophomonas* infection are fever and then cough [2]. However, in the present study, it was found that patients with symptoms of cough with sputum had the highest rate of *Lophomonas* infection, although these two symptoms may be without fever (17/47; 36.17%) or with fever (25/47; 36.17%). Therefore, fever may not be observed in all patients infected with the parasite, and this should be considered in the diagnosis.

## 5. Conclusions

To our knowledge, this is the first evidence about *Lophomonas* infection in patients suspected of having pulmonary tuberculosis in an endemic area. Given that the infection was relatively high in patients suspected of having tuberculosis and also had similar clinical symptoms of *Lophomonas* infection with tuberculosis, it is highly recommended that the sputum samples of these patients be examined for this parasite in order to make a correct diagnosis. Also, ensure that the patients receive timely treatment and the appropriate medication. Further investigations are needed to define the status of *Lophomonas* infection in patients suspected of having pulmonary tuberculosis in other areas of the world.

## Figures and Tables

**Table 1:** Demographic and clinical characteristics of patients suspected of having pulmonary tuberculosis, Golestan Province, northeastern Iran.

Variables	Description	No. of examined	No. of *Lophomonas*-positive (%)	No. of TB- positive (%)	No. of TB/*Lophomonas* coinfection (%)	*P* value
Gender	Male	99	24 (24.24)	5 (5.05)	0 (0)	0.51
	Female	117	23 (19.65)	4 (3.41)	2 (1.70)	

Age group	<20	12	2 (16.66)	0 (0)	0 (0)	0.57
	21–30	35	9 (25.71)	3 (8.57)	0 (0)	
	31–40	27	7 (25.92)	0 (0)	2 (7.41)	
	41–50	32	8 (25)	2 (6.25)	0 (0)	
	>50	110	21 (19.09)	4 (3.64)	0 (0)	

Clinical findings	Cough	188	38 (20.21)	9 (4.79)	2 (1.06)	0.84
	Sputum	179	39 (21.79)	8 (4.47)	1 (0.56)	
	Fever	72	17 (23.61)	5 (6.94)	0 (0)	

Place	Aq Qala	85	19 (22.35)	3 (3.53)	1 (1.18)	0.007
	Gonbad-e Kavus	103	4 (3.88)	6 (5.82)	1(0.97)	
	Gorgan	28	24 (85.71)	0 (0)	0 (0)	

Total		216	47 (21.76)	9 (4.16)	2 (0.92)	

## Data Availability

The data are available from the corresponding author upon reasonable request.

## References

[1] Chakaya J., Khan M., Ntoumi F. (2021). Global Tuberculosis Report 2020–Reflections on the Global TB burden, treatment and prevention efforts. *International Journal of Infectious Diseases*.

[2] Silva D. R., Mello F. CdQ., Migliori G. B. (2020). Tuberculosis series 2020. *Jornal Brasileiro de Pneumologia*.

[3] Tabrizi J. S., Rostami F. F., Ahmadi S. S., Dolatabad S. S. (2014). Socio-demographic factors affecting the prevalence of tuberculosis in Iran. *A General Policy*.

[4] Tavakoli A. (2017). Incidence and prevalence of tuberculosis in Iran and neighboring countries. *Zahedan Journal of Research in Medical Sciences*.

[5] Xue J., Li Y. L., Yu X. M. (2014 Oct). Bronchopulmonary infection of *Lophomonas blattarum*: a case and literature review. *Korean Journal of Parasitology*.

[6] Fakhar M., Sharifpour A., Nakhaei M., Banimostafavi E. S., Ghasemi M., Abedian S. (2021). *Lophomonas and Lophomoniasis*.

[7] Van Woerden H. C., Ratier-Cruz A., Aleshinloye O. B., Martinez-Giron R., Gregory C., Matthews I. P. (2011). Association between protozoa in sputum and asthma: a case-control study. *Respiratory Medicine*.

[8] Thakur C., Verma S., Negi R. S., Kumar V., Gupta S., Sharma V. (2017). *Lophomonas blattarum* co-infection in a patient with multidrug-resistant tuberculosis. *International Journal of Tuberculosis & Lung Disease*.

[9] Talebian M., Berenji F., Amini M. (2018). A study about clinical symptoms and laboratory signs of adult and pediatric patients with *Lophomonas blattarum*. *Journal of Research in Medical and Dental Science*.

[10] Ghafarian N., Bakhtiari E., Berenji F. (2018). The study of *Lophomonas blattarum* infection in children with respiratory symptoms: a descriptive clinical study in north east of Iran. *International Journal of Pediatrics*.

[11] Fakhar M., Nakhaei M., Sharifpour A. (2021). Morphological and Molecular Identification of Emerged *Lophomonas* Blattarum Infection in Mazandaran Province, Northern Iran: firstRegistry-Based Study. *Acta Parasitol*.

[12] Fakhar M., Nakhaei M., Sharifpour A. (2019). First molecular diagnosis of lophomoniasis: the end of a controversial story. *Acta Parasitologica*.

[13] Berenji F., Hosseini Farash B. R., Talebian M. (2021 Oct 1). Different staining methods in diagnosing *Lophomonas blattarum* in bronchoalveolar lavage samples. *Journal of Patient Safety & Quality Improvement*.

[14] Li R., Gao Z.-C. (2016). *Lophomonas blattarum* infection or just the movement of ciliated epithelial cells?. *Chinese Medical Journal*.

[15] Martinez-Girón R., Cornelis van Woerden H. (2013). *Lophomonas blattarum* and bronchopulmonary disease. *Journal of Medical Microbiology*.

[16] Failoc-Rojas V. E., Iglesias-Osores S., Silva-Díaz H. (2020). *Lophomonas* sp. in the upper and lower respiratory tract of patients from a hospital in Lambayeque, Peru: clinical case studies. *Respiratory medicine case reports*.

[17] Verma S., Verma G., Singh D. V. (2015). Dual infection with pulmonary tuberculosis and *Lophomonas blattarum* in India. *International Journal of Tuberculosis & Lung Disease*.

[18] Martínez-Girón R., Ribas A., Astudillo-González A. (2007). Flagellated protozoa in cockroaches and sputum: the unhygienic connection?. *Allergy and Asthma Proceedings*.

[19] Mirzazadeh F., Berenji F., Amini M. (2017). *Lophomonas blattarum* in asthmatic patients and control group. *Journal of Research in Medical and Dental Science*.

[20] Ding Q., Shen K. (2021 Jan). Pulmonary infection with *Lophomonas blattarum*. *Indian Journal of Pediatrics*.

